# Degalactosylated whey protein increases α-Klotho concentrations in the plasma and kidney in mice

**DOI:** 10.3389/fnut.2026.1839336

**Published:** 2026-06-24

**Authors:** Toshio Inui, Namiko Kawamura, Masahiro Yamamura, Rikako Yamamura, Noriko Satoh-Asahara, Yoshihiro Ogawa, Goro Katsuura

**Affiliations:** 1 Saisei Pharma, Moriguchi, Japan; 2 Department of Psychosomatic Internal Medicine, Kagoshima University Graduate School of Medical and Dental Sciences, Kagoshima, Japan; 3 Department of Medicine and Bioregulatory Science, Graduate School of Medical Sciences, Kyushu University, Fukuoka, Japan; 4 Osaka Medical and Pharmaceutical University, Takatsuki, Japan; 5 Department of Endocrinology, Metabolism, and Hypertension Research, Clinical Research Institute, NHO Kyoto Medical Center, Kyoto, Japan

**Keywords:** degalactosylated whey protein, kidney, mice, plasma, α-Klotho

## Abstract

With the acceleration of global population aging, maintaining healthy longevity in an aging society become an important concern. This study examined whether D-WP influences key factors of a healthy lifespan, such as α-Klotho, fibroblast growth factor-23 (FGF-23), and sirtuin 1 (SIRT1), in mice. Mice who received oral D-WP for 8 days exhibited significantly increased plasma α-Klotho concentrations. On the other hand, plasma concentrations of FGF-23 and SIRT1 were not changed by oral intake of D-WP. Moreover, kidney α-Klotho protein concentrations were also significantly increased by oral intake of D-WP for 8 days. Oral intake of intact whey protein did not induce the same effects as D-WP. These findings suggest that D-WP may have a potency to increase plasma Klotho levels.

## Introduction

Dietary whey protein has immunoregulatory and antioxidant effects (1, 2) and is thus recognized as a functional food with nutritional applications and health benefits (1, 3). Whereas protein glycosylation regulates multiple biological activities (4–6), protein deglycosylation converts biologically inactive proteins to biologically active proteins (7), suggesting that protein deglycosylation induces novel biological actions. Based on *in vivo* and *in vitro* experiments in mice, we previously demonstrated that degalactosylated whey protein (D-WP), but not intact whey protein (WP), potently prevents lipopolysaccharide-induced inflammation (8). Moreover, a recent report indicated that oral intake of D-WP, but not WP, significantly elongates peripheral blood telomere length in both young and aged mice (9). The present study examined whether D-WP regulates plasma longevity-related factors such as α-Klotho, fibroblast growth factor-23 (FGF-23), and sirtuin 1 (SIRT1) in mice.

Klotho was originally identified as an anti-aging protein in mice because mice genetically lacking Klotho develop various phenotypic features associated with premature aging (10). α-Klotho, generated through cleavage of the extracellular domain of the full-length Klotho protein by secretases, is found in blood, urine, and cerebrospinal fluid (11–13). α-Klotho is primarily expressed in the kidney, although lower levels of expression are detected in the brain and other peripheral organs (10, 14). Plasma α-Klotho concentrations inversely correlate with age in humans, non-human primates, and rodents (15–17). Moreover, mice with lower α*-Klotho* gene expression exhibit a shortened lifespan and premature onset of age-related phenotypes (10), while α*-Klotho* gene overexpression is associated with a prolonged lifespan (18). The full-length membrane-bound Klotho is a co-receptor for FGF-23 (19, 20). Several genetic studies in mice and humans have produced compelling evidence that Klotho is essential for FGF-23-mediated regulation of systemic phosphate homeostasis and vitamin D metabolism (19, 20). Aging-related kidney dysfunction is attributed to decreasing α-Klotho concentrations along with a subsequent decrease in FGF receptor function (21).

SIRT1 is a NAD^+^-dependent deacetylase linked to lifespan regulation in several species through its effects on energy metabolism (22). SIRT1 is also considered an anti-aging protein due to its protective effects against age-related diseases (23, 24). Furthermore, circulating concentrations of SIRT1 decline with aging (25).

In the present study, we examined the effects of orally ingested D-WP on the plasma concentrations of α-Klotho, FGF-23, and SIRT1, and moreover, α-Klotho concentrations in the kidney in young mice.

## Materials and methods

### Experimental animals

Male C57BL/6 J mice (2 months old) were obtained from CLEA Japan, Inc. (Tokyo, Japan) and housed in plastic cages under a 12:12 h light/dark cycle (lights turned on at 7 a.m.) at room temperature (23 ± 1 °C) with free access to water and food (CE-2; CLEA Japan, Inc.), and were used in the experiments at 3 months of age. Mice were randomly divided into three groups; water intake, D-WP intake, and WP intake. In another experiment to examine the protein levels of α-Klotho in the kidney, mice were randomly divided into three groups; water intake, D-WP intake, and WP intake. All animal experiments complied with the ARRIVE guidelines and were performed in accordance with the guidelines established by the Institute of Laboratory Animal Science Research Support Center at Kagoshima University. The animal experiments were approved by the Kagoshima University Institutional Animal Care and Use Committee (protocol nos. MD15060, MD16052, MD17060, and MD18079), and in accordance with the guidelines established by the United States National Institutes of Health Guide for the Care and Use of Laboratory Animals (NIH publication No. 80–23, revised in 1996). Every effort was made to minimize the number of animals used and to optimize their comfort.

### Preparation of D-WP

Whey protein was obtained from Yotsuba Milk Products Co., Ltd. (Sapporo, Japan). The D-WP was prepared by Saisei Pharma (Moriguchi, Japan) according to our previous report (9, 26). Briefly, 1 mg of whey protein was dissolved in 1 mL of 50 mM sodium phosphate buffer (pH 7.0) and incubated with 65 mU of *β*-D-galactosidase (from *Escherichia coli*; WAKO Pure Chemical Industries, Ltd., Osaka, Japan) at 37 °C for 1 h. The reaction mixture was heated at 60 °C for 10 min to inactivate the enzyme. The protein concentrations were determined using a Pierce^®^ BCA protein assay kit (Thermo Fisher Scientific Inc., Waltham, MA, United States).

### Experimental schedule

Whey protein mixed with drinking water in water bottle at the concentration of 60 μg/mL was given to mice for 8 days. Whey protein-mixed water was freshly prepared and changed every 2 days. Water was given to mice as a control.

At the end of the experiments, blood samples were collected in tubes containing EDTA (10 μL of 0.2 M EDTA/tube) from the retroorbital vein under 1.0% isoflurane (FUJIFILM Wako Pure Chemical Corporation, Osaka, Japan) anesthesia using an anesthesia apparatus for small animals (MK-AT210D, Muromachi Kikai Co., Ltd., Osaka, Japan) between 1,000 and 1,200 h (27). The blood samples were centrifuged at 3000xg for 5 min at 4 °C, and plasma was separated and stored at −80 °C until assayed. After blood sampling, the mice were killed by decapitation, and the kidney was rapidly dissected, frozen in liquid nitrogen, and stored at −80 °C until assayed (28).

### Measurement of protein concentrations of α-klotho, SIRT1, and FGF-23

Plasma concentrations of α-Klotho, SIRT1, and FGF-23 were measured in duplicate with 100-μL samples using commercially available measurement kits, Mouse soluble α-Klotho Assay Kit – IBL (Immuno-Biological Laboratories Co., Ltd., Fujioka, Japan), mouse FGF23 ELISA Kit (ab213863; Abcam Inc., Cambridge, United Kingdom), and mouse SIRT1 ELISA Kit (ab206983; Abcam Inc.), respectively.

The protein concentrations of α-Klotho in the kidney were measured using commercially available ELISA kit. Before application of measurement, the kidney samples were prepared according to the previous report (29). The frozen kidney regions were homogenized with N-PER Neuronal Protein Extraction Reagent (Thermo Fisher Scientific Inc.) containing Phosphatase Inhibitor Cocktails 2, 3 and a Protease Inhibitor Cocktail (MilliporeSigma, St. Louis, MO, United States). Supernatants were collected after centrifugation at 13,000 xg for 10 min at 4 °C, and protein concentrations were determined using a Pierce^®^ BCA protein assay kit (Thermo Fisher Scientific Inc.). The protein concentrations of α-Klotho in the kidney samples were determined in duplicate with 100-μL samples using commercially available measurement kits, Mouse soluble α-Klotho Assay Kit – IBL (Immuno-Biological Laboratories Co., Ltd.). The protein concentrations of α-Klotho in the kidney were expressed per milligram protein concentration.

### Statistical analysis

Data are expressed as mean ± SEM. Statistical analysis of the data was performed by ANOVA followed by the Tukey–Kramer test. Statistical significance was defined as *p* < 0.05.

## Results

### Effects of D-WP on plasma α-klotho, FGF-23, and SIRT1 concentrations in mice

**Figure 1:**
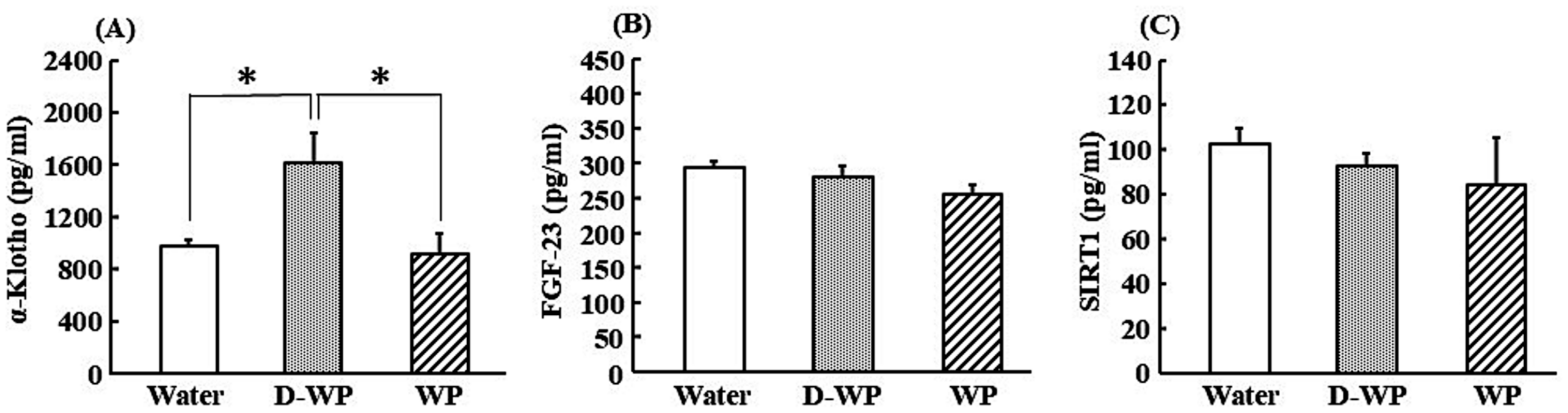
Effects of D-WP intake for 8 days on plasma α-Klotho, FGF-23, and SIRT1 concentrations in mice. Plasma concentrations (pg/mL) of **(A)** α-Klotho, **(B)** FGF-23, and **(C)** SIRT1 were examined in mice following oral intake of D-WP and WP for 8 days. Results are expressed as mean ± SEM for 5–16 mice. *p < 0.05.

Oral intake of D-WP for 8 days significantly increased plasma α-Klotho concentrations to 166% of those in control mice [*F*_(2, 39)_ = 5.594, *p* < 0.01], whereas oral intake of WP had no effect on plasma α-Klotho concentrations (Figure 1A). Neither D-WP nor WP affected plasma FGF-23 and SIRT1 concentrations [*F*_(2, 26)_=2.814, *p* > 0.05] and [*F*_(2, 21)_=0.770, *p* > 0.05], respectively (Figures 1B,C).

### Effects of D-WP on the α-klotho protein concentration in the kidney in mice

**Figure 2:**
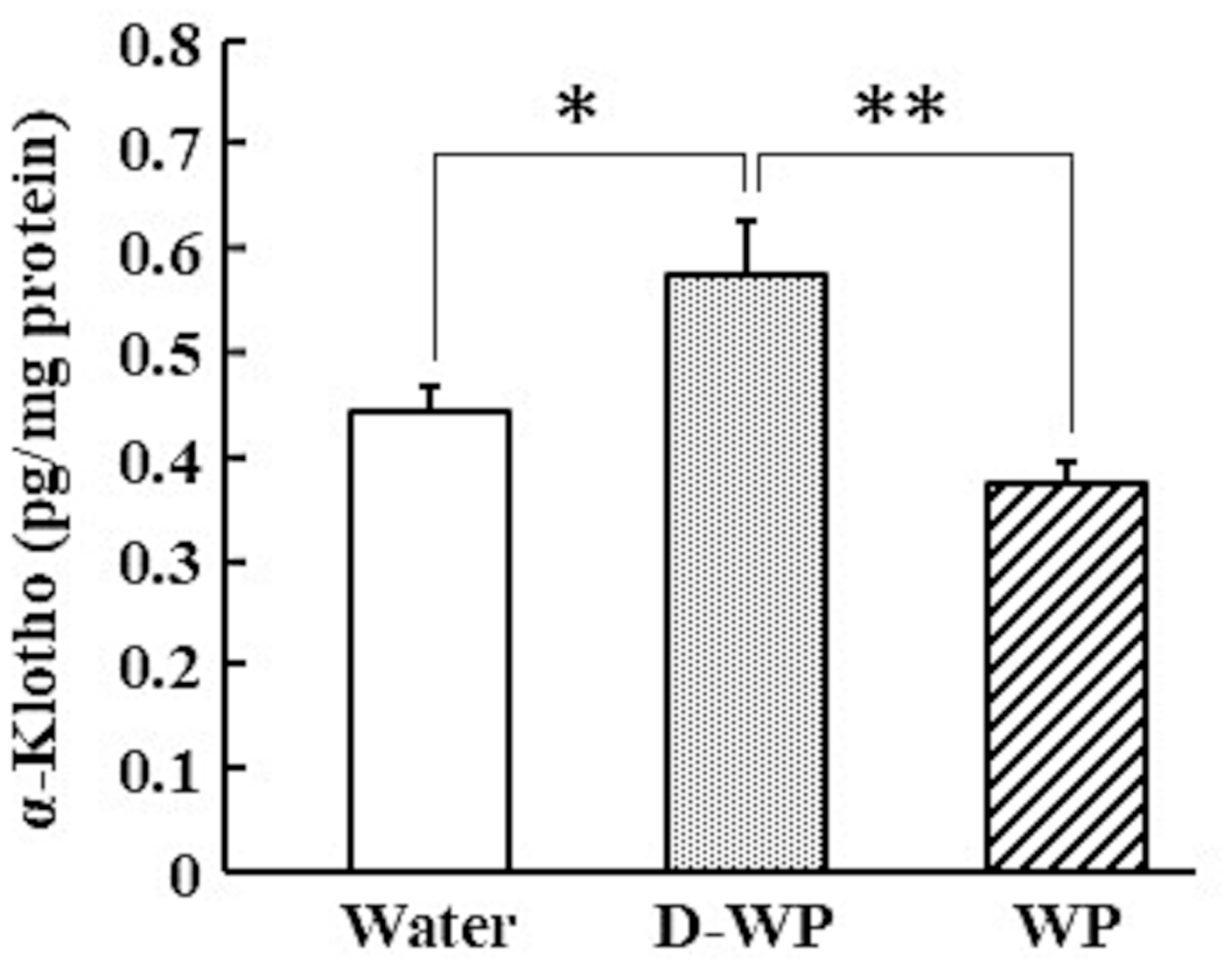
Effects of D-WP intake for 8 days on the α-Klotho concentration in mouse kidney. The α-Klotho concentration (pg/mg protein) was examined in mouse kidney following oral intake of D-WP and WP for 8 days. Results are expressed as mean ± SEM for 8 mice. *p < 0.05, **p < 0.01.

The α-Klotho protein concentration in the kidney significantly increased to 130% of that in control mice following oral intake of D-WP for 8 days [*F*_(2, 21)_=8.162, *p* < 0.01] (Figure 2). Oral intake of WP had no effect on the α-Klotho protein concentration in the kidney (Figure 2).

## Discussion

The present study, examining the effects of D-WP on longevity-related factors, demonstrated that D-WP, but not WP, significantly increased plasma α-Klotho concentrations and α-Klotho protein concentration in the kidney following oral intake for 8 days in mice. This study provided preliminary evidence for a novel function for D-WP as an increasing regulator of Klotho levels.

Previous *in vivo* and *in vitro* experiments in mice showed that D-WP, but not WP, potently suppresses lipopolysaccharide-induced inflammation (8). Another recent report demonstrated that oral intake of D-WP, but not WP, significantly elongates peripheral blood telomere length along with an increase in telomerase in young and aged mice (9). Together, these findings highlight the novel actions of D-WP compared with WP.

Profound anti-aging activities of α-Klotho are reported in both animal and human studies (30, 31). α-Klotho is primarily expressed in the kidney and the choroid plexus in the brain (10, 18). Klotho exists in three forms, each with unique functions: full-length transmembrane Klotho (FL-KL), shed Klotho (shKL), and secreted Klotho (α-Klotho), which is produced via differential splicing (11, 21). Plasma α-Klotho concentrations are consistently found to inversely correlate with age in humans, non-human primates, and rodents (15). Noteworthy, mice with lower α-*Klotho* gene expression have a shortened lifespan and premature onset of age-related phenotypes observed in association with human aging, including arteriosclerosis, osteoporosis, cognitive decline, and neurodegeneration of hippocampal and cerebellar Purkinje neurons (10). α-*Klotho*-deficient mice exhibit profoundly impaired cognition (32). In contrast, α-*Klotho* gene overexpression prolongs lifespan (33). These findings clearly indicate that α-Klotho is essential for normal higher brain function and cognitive performance (34). Interestingly, peripheral administration of α-Klotho protein fragments enhanced cognition and neural resilience in young, aging, and transgenic α-synuclein mice, a neurodegenerative disease model, despite the inability of these fragments to cross the blood–brain barrier, acting through the NMDAR subunit GluN2B (35).

In the kidney, FL-KL and shKL function as co-receptors with FGFR1 for FGF-23 signaling (36). FGF-23 exerts its biological effects by activating FGFRs in an α-Klotho-dependent manner (37), which regulate the concentrations of circulating calcium, phosphate, and vitamin D (36). Therefore, it is possible that D-WP indirectly influences these FGFR-mediated actions. Because the kidney is the main source of plasma α-Klotho (10), D-WP intake may increase the α-Klotho concentration in the kidney, resulting in higher plasma α-Klotho concentrations. Further experiments are necessary to determine the mechanistic and functional evidence supporting the anti-aging and improvement of cognition via increased Klotho levels. Since, regarding measurement of human serum Klotho levels, it was reported that immunoprecipitation-immunoblot assay is superior to ELISA (38), it should be evaluated. More importantly, the 8-day treatment period use in the present study is too short to support strong conclusions regarding long-term physiological effects and aging biology. In addition, since the experiments were performed only in young mice, the experiment using aged mice should be necessary to explore the translational relevance of this finding. As such, because the present study just showed the preliminary results, further examination should be conducted.

In the present study, we demonstrated that oral intake of D-WP, but not WP, significantly increases α-Klotho concentrations in the plasma and kidney in mice. This study revealed the novel action of D-WP.

## Data Availability

The original contributions presented in the study are included in the article/supplementary material, further inquiries can be directed to the corresponding authors.
